# Supplementary material for: Beyond Harmful: Exploring Biofilm Formation by Enterococci Isolated from Portuguese Traditional Cheeses
Source: Foods. 2024 Sep 26;13(19):3067. doi: 10.3390/foods13193067 (PMC11476095; doi:10.3390/foods13193067)
Supplement: Supplementary file 1 [file foods-13-03067-s001.zip › Supplementary Figures.pdf]

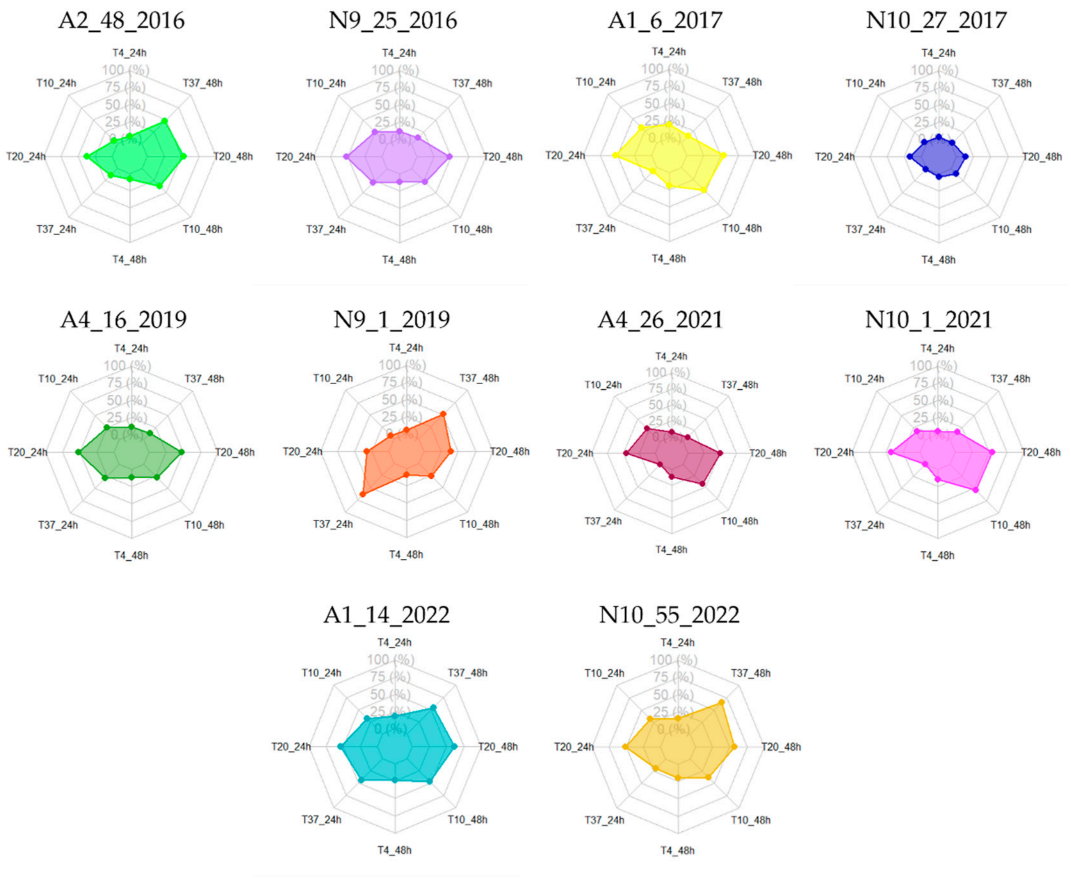

Supplementary Figure S1 - Cell viability at different temperatures (4, 10, 20 and 37 °C) and incubation periods (24 and 48 h). Legend: A1-A5 – *Azeitão* cheese factories; N9-N10 – *Nisa* cheese factories. The scale used for the graphs represents cell viability through quantification of reduced resazurin (in percentage).

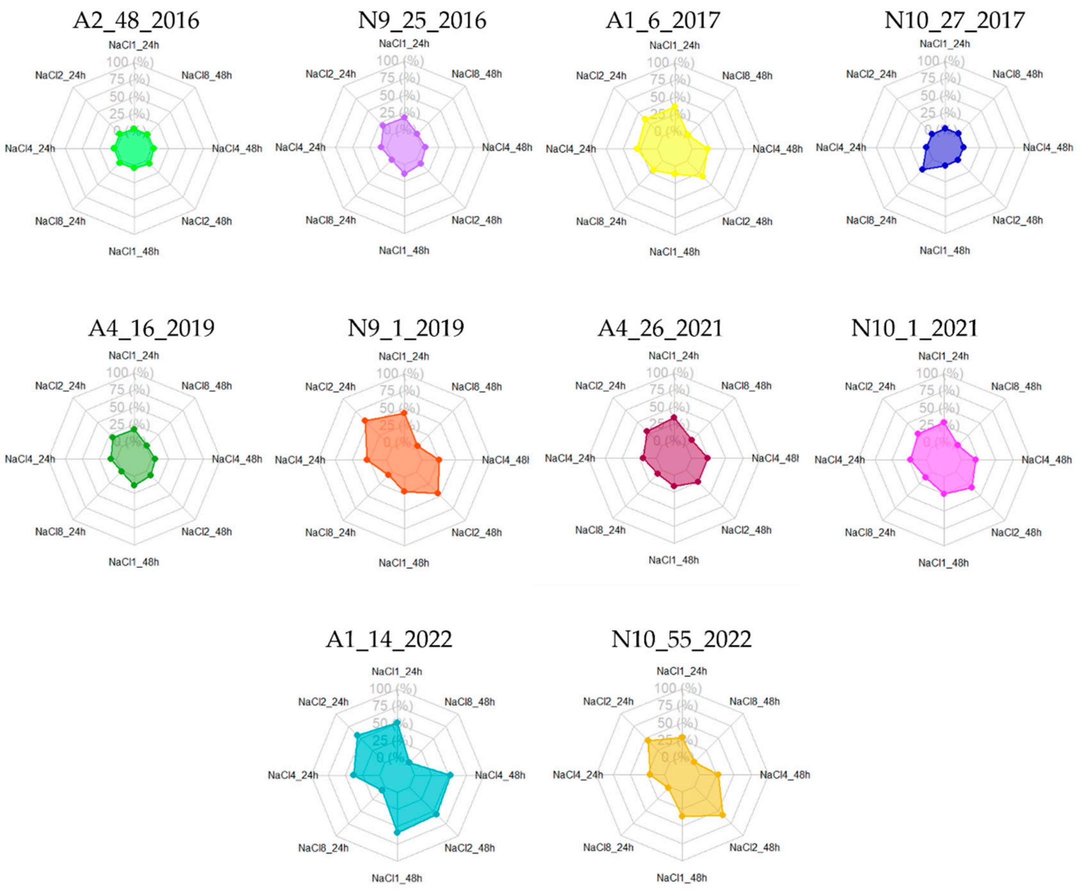

Supplementary Figure S2 - Cell viability at NaCl concentrations (1, 2, 4 and 8 %) and incubation periods (24 and 48 h). Legend: A1-A5 – *Azeitão* cheese factories; N9-N10 – *Nisa* cheese factories. The scale used for the graphs represents cell viability through quantification of reduced resazurin (in percentage).

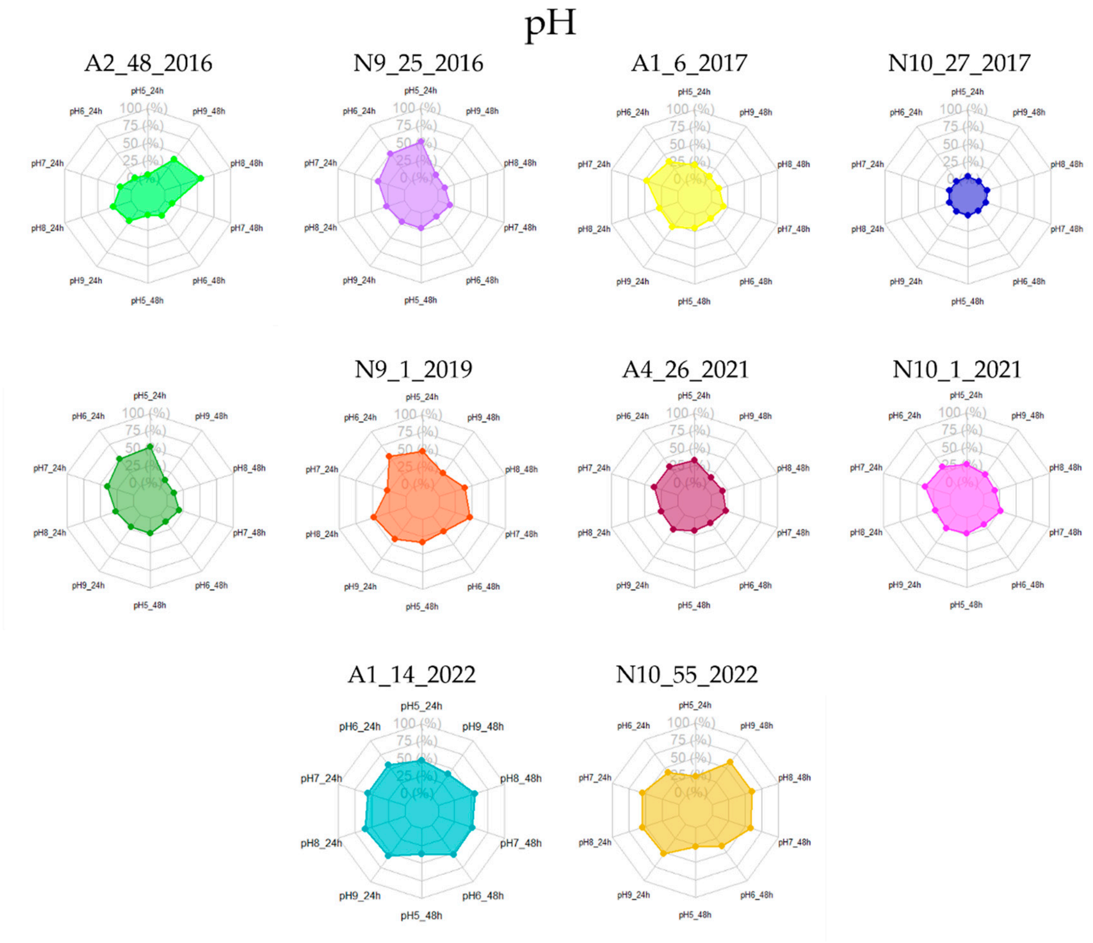

Supplementary Figure S3 - Cell viability at different pH values (5, 6, 7, 8 and 9) and incubation periods (24 and 48 h). Legend: A1-A5 – *Azeitão* cheese factories; N9-N10 – *Nisa* cheese factories. The scale used for the graphs represents cell viability through quantification of reduced resazurin (in percentage).

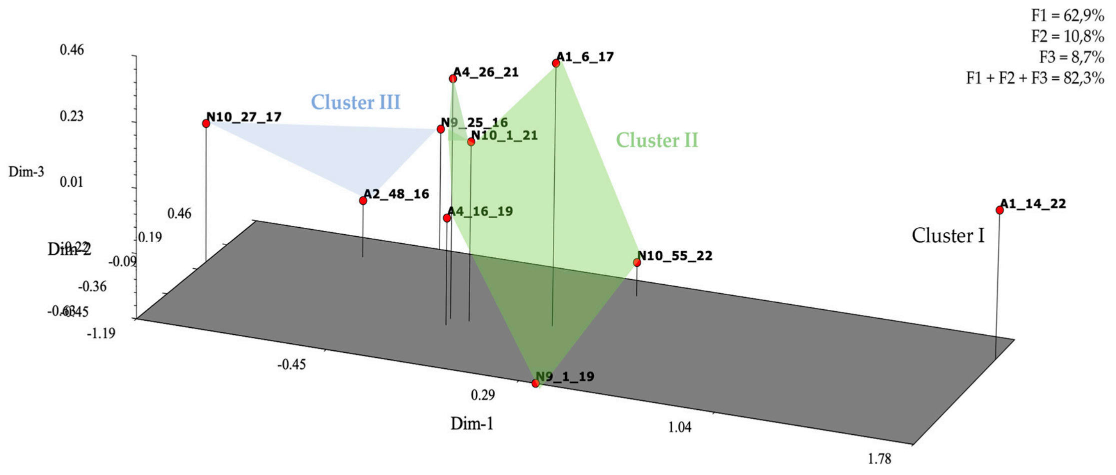

Supplementary Figure S4 - Three-dimensional diagram obtained after PCA. Legend: A - *Azeitão* cheese factories; N – *Nisa* cheese factories; Dim-1 – First principal component; Dim-2 – Second principal component; Dim-3 – Third principal component; F1 – Percentage of variance of the first principal component; F2 – Percentage of variance of the second principal component; F3 – Percentage of variance of the third principal component; F1+F2+F3 – Percentage of the total variance of the three principal components.
