# Supplementary material for: Beyond Harmful: Exploring Biofilm Formation by Enterococci Isolated from Portuguese Traditional Cheeses
Source: Foods. 2024 Sep 26;13(19):3067. doi: 10.3390/foods13193067 (PMC11476095; doi:10.3390/foods13193067)
Supplement: Supplementary file 1 [file foods-13-03067-s001.zip › Supplementary Tables.pdf]

Supplementary Table S1 - *Enterococcus* spp. from PDO cheeses produced in Azeitão and Nisa between 2016-22.

Isolate	Identification	Year of production	Region	Cheese factory
A1.40.2016	*E. faecalis*	2016	*Azeitão*	A1
A2.48.2016	*E. durans*	2016	*Azeitão*	A2
A3.34.2016	*E. faecium*	2016	*Azeitão*	A3
A4.19.2016	*E. durans*	2016	*Azeitão*	A4
A5.27.2016	*E. durans*	2016	*Azeitão*	A5
N9.25.2016	*E. faecium*	2016	*Nisa*	N9
N10.46.2016	*E. faecalis*	2016	*Nisa*	N10
A1.6.2017	*E. durans*	2017	*Azeitão*	A1
A2.17.2017	*E. durans*	2017	*Azeitão*	A2
A3.9.2017	*E. faecalis*	2017	*Azeitão*	A3
A4.6.2017	*E. faecium*	2017	*Azeitão*	A4
A5.18.2017	*E. faecalis*	2017	*Azeitão*	A5
N9.10.2017	*E. faecium*	2017	*Nisa*	N9
N10.27.2017	*E. faecium*	2017	*Nisa*	N10
A1.9.2019	*E. faecalis*	2019	*Azeitão*	A1
A2.20.2019	*E. faecium*	2019	*Azeitão*	A2
A3.2.2019	*E. faecium*	2019	*Azeitão*	A3
A4.16.2019	*E. faecalis*	2019	*Azeitão*	A4
N9.1.2019	*E. faecalis*	2019	*Nisa*	N9
N10.14.2019	*E. durans*	2019	*Nisa*	N10
A1.2.2021	*E. faecalis*	2021	*Azeitão*	A1
A1.24.2021	*E. faecalis*	2021	*Azeitão*	A1
A1.45.2021	*E. durans*	2021	*Azeitão*	A1
A1.59.2021	*E. faecalis*	2021	*Azeitão*	A1
A2.3.2021	*E. faecium*	2021	*Azeitão*	A2
A2.17.2021	*E. durans*	2021	*Azeitão*	A2

A2.32.2021	*E. faecium*	2021	*Azeitão*	A2
A2.58.2021	*E. faecalis*	2021	*Azeitão*	A2
A3.4.2021	*E. faecalis*	2021	*Azeitão*	A3
A3.9.2021	*E. durans*	2021	*Azeitão*	A3
A3.16.2021	*E. faecalis*	2021	*Azeitão*	A3
A3.19.2021	*E. faecalis*	2021	*Azeitão*	A3
A3.26.2021	*E. faecalis*	2021	*Azeitão*	A3
A3.39.2021	*E. faecalis*	2021	*Azeitão*	A3
A3.45.2021	*E. faecalis*	2021	*Azeitão*	A3
A4.7.2021	*E. faecalis*	2021	*Azeitão*	A4
A4.11.2021	*E. faecalis*	2021	*Azeitão*	A4
A4.21.2021	*E. faecalis*	2021	*Azeitão*	A4
A4.26.2021	*E. faecalis*	2021	*Azeitão*	A4
A4.41.2021	*E. faecalis*	2021	*Azeitão*	A4
N9.21.2021	*E. durans*	2021	*Nisa*	N9
N9.46.2021	*E. durans*	2021	*Nisa*	N9
N9.58.2021	*E. faecalis*	2021	*Nisa*	N9
N10.1.2021	*E. faecalis*	2021	*Nisa*	N10
N10.21.2021	*E. faecalis*	2021	*Nisa*	N10
N10.45.2021	*E. faecalis*	2021	*Nisa*	N10
N10.47.2021	*E. faecalis*	2021	*Nisa*	N10
N10.49.2021	*E. faecalis*	2021	*Nisa*	N10
A1.14.2022	*E. faecalis*	2021	*Azeitão*	A1
A1.54.2022	*Enterococcus* sp.	2022	*Azeitão*	A1
A2.1.2022	*E. durans*	2022	*Azeitão*	A2
A2.23.2022	*E. faecalis*	2022	*Azeitão*	A2
A2.34.2022	*E. faecalis*	2022	*Azeitão*	A2
A2.38.2022	*E. faecalis*	2022	*Azeitão*	A2
A3.14.2022	*E. durans*	2022	*Azeitão*	A3
A3.38.2022	*E. faecalis*	2022	*Azeitão*	A3
A3.47.2022	*E. faecalis*	2022	*Azeitão*	A3

A3.49.2022	*E. faecalis*	2022	*Azeitão*	A3
A3.57.2022	*E. faecalis*	2022	*Azeitão*	A3
A4.5.2022	*E. faecalis*	2022	*Azeitão*	A4
A4.11.2022	*E. durans*	2022	*Azeitão*	A4
A4.12.2022	*E. faecalis*	2022	*Azeitão*	A4
A4.20.2022	*E. durans*	2022	*Azeitão*	A4
A4.30.2022	*E. faecalis*	2022	*Azeitão*	A4
A4.43.2022	*E. faecalis*	2022	*Azeitão*	A4
A4.54.2022	*E. faecalis*	2022	*Azeitão*	A4
A4.59.2022	*E. faecalis*	2022	*Azeitão*	A4
N9.1.2022	*E. faecalis*	2022	*Nisa*	N9
N9.23.2022	*E. faecalis*	2022	*Nisa*	N9
N9.58.2022	*E. faecalis*	2022	*Nisa*	N9
N10.18.2022	*E. faecalis*	2022	*Nisa*	N10
N10.55.2022	*E. faecalis*	2022	*Nisa*	N10

Legend: A –*Azeitão* ; N – *Nisa*. A1-A5 – *Azeitão* cheese factories; N9-N10 – *Nisa* cheese factories.

Supplementary Table S2 – Statistical analysis of biofilm production ratio of the enterococci isolates for two incubation periods 24 and 48 h after crystal viotel staining.

**24 vs. 48 h 37 °C – Crystal violet**							
p- values							
**Isolates**		**Isolates**		**Isolates**		**Isolates**	
A1.40 2016	$p < 0.05$	N9.1 2019	N.S	A4.11 2021	N.S	A3.14 2022	$p < 0.05$
A2.48 2016	$p < 0.05$	N10.14 2019	$p < 0.05$	A4.21 2021	N.S	A3.38 2022	N.S
A3.34 2016	$p < 0.05$	A1.2 2021	N.S	A4.26 2021	$p < 0.05$	A3.47 2022	N.S
A4.19 2016	$p < 0.05$	A1.24 2021	$p < 0.05$	A4.41 2021	N.S	A3.49 2022	N.S
A5.17 2016	N.S	A1.45 2021	N.S	N9.21 2021	N.S	A3.57 2022	N.S
N9.25 2016	N.S	A1.59 2021	N.S	N9.46 2021	N.S	A4.5 2022	N.S
N10.46 2016	N.S	A2.3 2021	N.S	N9.58 2021	$p < 0.05$	A4.11 2022	N.S
A1.6 2017	$p < 0.05$	A2.17 2021	N.S	N10.1 2021	N.S	A4.12 2022	N.S
A2.17 2017	$p < 0.05$	A2.32 2021	N.S	N10.21 2021	$p < 0.05$	A4.20 2022	N.S
A3.9 2017	N.S	A2.58 2021	N.S	N10.45 2021	$p < 0.05$	A4.30 2022	N.S
A4.6 2017	N.S	A3.4 2021	N.S	N10.47 2021	N.S	A4.43 2022	N.S
A5.18 2017	$p < 0.05$	A3.9 2021	N.S	N10.49 2021	N.S	A4.54 2022	N.S
N9.1 2017	N.S	A3.16 2021	N.S	A1.14 2022	N.S	A4.59 2022	N.S
N10.27 2017	N.S	A3.19 2021	N.S	A1.54 2022	N.S	N9.1 2022	N.S
A1.9 2019	N.S	A3.26 2021	N.S	A2.1 2022	N.S	N9.23 2022	N.S
A2.20 2019	$p < 0.05$	A3.39 2021	N.S	A2.23 2022	N.S	N9.58 2022	N.S
A3.2 2019	$p < 0.05$	A3.45 2021	N.S	A2.34 2022	N.S	N10.18 2022	N.S
A4.16 2019	N.S	A4.7 2021	N.S	A2.38 2022	N.S	N10.55 2022	N.S

Legend: N.S: - non-signifficant; A –*Azeitão* ; N – *Nisa*. A1-A5 – *Azeitão* cheese factories; N9-N10 – *Nisa* cheese factories

Supplementary Table S3 – Correlation coefficients between the original variables and the principal components.

Crystal violet variable	Dim-1	Dim-2	Dim-3
4 °C 24 h	0.8723	0.3341	0.0825
10 °C 24 h	0.8532	0.3024	0.1950
20 °C 24 h	0.7912	0.4518	0.1679
37 °C 24 h	0.6813	-0.4222	0.4806
4 °C 48 h	0.6990	0.5945	0.0447
10 °C 48 h	0.8287	0.3634	0.2397
20 °C 48 h	0.8118	-0.3114	-0.1038
37 °C 48 h	0.8587	0.1378	-0.1172
NaCl 1 % 24 h	0.9312	-0.2906	0.1310
NaCl 2 % 24 h	0.9420	-0.2591	-0.0265
NaCl 4 % 24 h	0.9161	-0.1600	0.1297
NaCl 8 % 24 h	0.6719	0.2260	0.5448
NaCl 1 % 48 h	0.9675	0.0937	0.0608
NaCl 2 % 48 h	0.9679	0.0209	0.1398
NaCl 4 % 48 h	0.9486	0.0936	0.1063
NaCl 8 % 48 h	0.6321	-0.0472	0.6045
pH 5 24 h	0.7951	-0.5470	-0.0917
pH 6 24 h	0.8499	-0.4708	-0.0341
pH 7 24 h	0.6170	-0.5816	-0.4250
pH 8 24 h	0.8967	-0.1117	-0.0623
pH 9 24 h	0.9239	0.2112	-0.0698
pH 5 48 h	0.7352	-0.5977	0.0615
pH 6 48 h	0.9262	-0.1861	0.0916
pH 7 48 h	0.9323	0.1093	-0.0107
pH 8 48 h	0.8746	0.3265	-0.0169
pH 9 48 h	0.8734	0.3336	-0.0410
**Resazurin variable**	**Dim-1**	**Dim-2**	**Dim-3**
4 °C 24 h	0.7460	0.1634	0.2639
10 °C 24 h	0.6527	0.0567	0.4344
20 °C 24 h	0.5805	0.3295	0.2330
37 °C 24 h	0.6012	-0.3555	-0.6202
4 °C 48 h	0.6549	0.5292	0.2319
10 °C 48 h	0.5451	0.1896	0.4466
20 °C 48 h	0.6237	0.3372	0.1123
37 °C 48 h	0.5550	0.3067	-0.6929
NaCl 1 % 24 h	0.8851	-0.3221	0.1524

NaCl 2 % 24 h	0.8918	-0.2226	-0.1208
NaCl 4 % 24 h	0.8886	-0.2063	0.1963
NaCl 8 % 24 h	-0.3926	0.2501	0.4149
NaCl 1 % 48 h	0.9290	0.1056	-0.0687
NaCl 2 % 48 h	0.8524	-0.0243	-0.1563
NaCl 4 % 48 h	0.9438	-0.0007	0.1088
NaCl 8 % 48 h	-0.3625	-0.2982	0.5366
pH 5 24 h	0.6780	-0.5696	-0.0796
pH 6 24 h	0.8183	-0.4740	-0.0753
pH 7 24 h	0.8392	0.0691	0.1676
pH 8 24 h	0.7831	0.2569	-0.4120
pH 9 24 h	0.8900	0.1282	-0.2758
pH 5 48 h	0.8735	-0.3967	-0.1218
pH 6 48 h	0.9324	0.0714	-0.2180
pH 7 48 h	0.8241	-0.0335	-0.3965
pH 8 48 h	0.4394	0.5500	- 0.5953
pH 9 48 h	0.4100	0.6102	-0.5363

Legend: Dim-1 – first principal component; Dim-2 - second principal compenent; Dim-3 – third principal component; Green color indicates the explanatory variable for each principal component.

Supplementary Table S4 – Biofilm *per* cell.

	CV units/RZ units										
Assay	A2.48. 2016	N9.25. 2016	A1.6. 2017	N10.27. 2017	A4.16. 2019	N9.1. 2019	A4.26. 2021	N10.1. 2021	A1.14. 2022	N10.55. 2022	AV
4 °C at 24 h	0.73	1.16	0.97	0.28	0.58	0.81	0.32	0.25	3.55	1.26	0.99
10 °C at 24 h	0.73	0.66	0.53	0.31	0.19	0.44	0.57	0.20	2.19	0.54	0.64
20 °C at 24 h	0.55	0.44	0.46	0.12	0.40	0.19	0.30	0.36	1.17	0.37	0.44
37 °C at 24 h	0.75	1.74	12.82	2.86	1.10	1.25	0*	0*	2.37	1.47	2.44
4 °C at 48 h	0.65	1.84	0.83	0.24	0.45	0.45	0.42	0.26	2.85	1.16	0.91
10 °C at 48 h	0.21	0.59	0.39	0.16	0.17	0.21	0.52	0.53	1.73	0.60	0.51
20 °C at 48 h	0.35	0.29	0.51	0.12	0.89	0.90	0.42	0.55	0.74	0.46	0.52
37 °C at 48 h	0.66	0.64	2.06	2.50	7.40	0.98	2.41	1.63	3.71	1.01	2.30
1 % NaCl at 24 h	2.37	0.69	0.96	3.30	1.62	0.90	0.85	0.73	1.18	0.77	1.34
2 % NaCl at 24 h	2.54	0.63	0.91	2.29	1.81	0.73	0.69	1.04	1.02	0.62	1.23
4 % NaCl at 24 h	3.16	0.81	0.99	1.72	1.49	1.12	1.01	0.76	1.42	0.65	1.31
8 % NaCl at 24 h	2.08	1.04	1.90	0.83	8.83	2.34	5.59	1.59	10.05	5.94	4.02
1 % NaCl at 48 h	2.97	2.63	4.17	4.67	2.23	1.99	1.59	1.22	1.95	1.46	2.49
2 % NaCl at 48 h	1.37	1.00	1.41	6.17	2.21	0.82	0.89	1.00	1.56	0.60	1.70
4 % NaCl at 48 h	1.80	1.00	2.03	2.45	2.97	1.27	0.79	0.88	1.88	1.36	1.64
8 % NaCl at 48 h	5.75	7.94	35.06	2.93	6.31	15.49	8.41	8.14	0*	0*	9.0
pH 5 at 24 h	2.07	1.26	1.13	1.60	0.43	1.01	0.71	0.99	0.94	0.70	1.08
pH 6 at 24 h	1.90	1.02	0.76	2.41	0.56	0.85	0.68	0.77	0.93	0.38	1.03
pH 7 at 24 h	0.75	0.46	0.58	1.93	0.79	4.17	0.69	0.68	1.19	0.57	1.18
pH 8 at 24 h	0.77	0.56	1.10	2.04	1.30	0.79	0.86	1.15	0.93	0.41	0.99
pH 9 at 24 h	1.13	0.86	1.03	2.91	1.56	0.97	0.92	0.96	2.46	0.95	1.38
pH 5 at 48 h	3.72	4.26	2.33	4.44	1.32	1.91	1.78	2.13	1.45	0.97	2.43

pH 6 at 48 h	1.65	2.75	2.84	12.02	2.38	1.59	1.39	2.37	1.42	0.69	2.91
pH 7 at 48 h	1.62	1.21	2.27	3.61	2.20	1.08	1.02	1.17	3.07	0.77	1.80
pH 8 at 48 h	0.54	1.02	3.07	1.62	4.33	0.93	1.40	1.96	4.00	1.10	2.00
pH 9 at 48 h	0.59	0.62	3.07	2.95	4.32	1.05	1.36	1.68	6.87	1.75	2.43

Legend: * identifies isolates with non-detectable viability. The dark green color identifies which conditions induced higher biofilm production. CV – crystal violet; RZ – resazurin; AV – average.
